# Une triade diagnostique complexe mimant un syndrome d’activation macrophagique: leucémie lymphoïde chronique et tuberculose (à propos d’un cas)

**DOI:** 10.11604/pamj.2025.52.181.49806

**Published:** 2025-12-24

**Authors:** Rime Laassar, Ilham Orchi, Khadija Es-Sahli, Hafid Zahid

**Affiliations:** 1 Laboratoire d’Hématologie, Hôpital Militaire d’Instruction Mohammed V, Rabat, Maroc,; 2 Faculté de Médecine et de Pharmacie de Rabat, Rabat, Morocco

**Keywords:** Syndrome d’activation macrophagique, leucémie lymphoïde chronique, tuberculose pleurale, diagnostic, cas clinique, Macrophage activation syndrome, chronic lymphocytic leukemia, pleural tuberculosis, diagnosis, case report

## Abstract

Le syndrome d'activation macrophagique (SAM) est une entité rare potentiellement mortelle. Également connu sous le nom d'activation lympho-histiocytaire, son diagnostic repose sur la présence d'au moins 5 critères cliniques et biologiques. Le myélogramme vient apporter la confirmation du diagnostic en objectivant la présence de nombreux macrophages activés avec de multiples images d'hémophagocytose. Nous insistons à travers le cas d'un patient immunodéprimé suivi pour leucémie lymphoïde chronique (LLC) sur la difficulté de poser le diagnostic de SAM ainsi que sur la nécessité de dépister une tuberculose sous-jacente pouvant être masquée par les manifestations du syndrome lymphoprolifératif. Le diagnostic de SAM a été retenu sur la base d'anomalies cliniques, biologiques et cytologiques, avec une tuberculose pleurale identifiée comme facteur déclenchant. Ce cas souligne l’importance de prendre en compte les infections opportunistes et de maintenir une vigilance diagnostique chez les patients immunodéprimés, en mettant en lumière une triade clinique et épidémiologique inhabituelle.

## Abstract (en)

Abstract Macrophage activation syndrome (MAS) is a rare and potentially fatal condition. Also known as macrophage activation syndrome, its diagnosis is based on the presence of at least five clinical and biological criteria. Bone marrow examination helps confirm the diagnosis by demonstrating the presence of numerous activated macrophages with multiple hemophagocytosis images. Through the case of an immunocompromised patient followed for chronic lymphocytic leukemia (CLL), we emphasize the difficulty of diagnosing MAS and the need to screen for underlying tuberculosis, which may be masked by manifestations of the lymphoproliferative syndrome. The diagnosis of MAS was established based on clinical, biological, and cytological abnormalities, with pleural tuberculosis identified as the triggering factor. This case emphasizes the need to consider opportunistic infections and to maintain a high index of suspicion in immunocompromised patients, illustrating an unusual clinical and epidemiological triad. Keywords: macrophage activation syndrome; chronic lymphocytic leukemia; pleural tuberculosis; diagnosis; treatment.

## Introduction

Le syndrome d'activation macrophagique (SAM), ou hémophagocytose lymphohistiocytaire secondaire, est une hyperinflammation systémique potentiellement létale, caractérisée par une activation excessive des macrophages et des lymphocytes T, entraînant cytopénies, fièvre et atteinte multiviscérale [1]. Bien que rare, le SAM peut survenir secondairement à des hémopathies, des infections ou des maladies auto-immunes. La leucémie lymphoïde chronique (LLC), la leucémie adulte la plus fréquente, touche majoritairement les adultes de plus de 60 ans et prédispose aux infections opportunistes [2]. La tuberculose pleurale, manifestation extrapulmonaire courante, est observée chez 4-6% des patients tuberculeux [3]. La coexistence de SAM, LLC et tuberculose pleurale est extrêmement rare et pose un défi diagnostique majeur, en raison de la similitude des symptômes et anomalies biologiques [4,5]. Nous rapportons un cas illustrant cette triade clinique et épidémiologique exceptionnelle, afin de sensibiliser les cliniciens à cette association et à la nécessité d'une prise en charge rapide et adaptée.

## Méthodes

**Présentation du patient:** patient âgé de 60 ans, sous surveillance pour une leucémie lymphoïde chronique (LLC) stade B, depuis trois ans, traité pour une hépatite B, consulte aux urgences pour une détresse respiratoire avec syndrome d'épanchement pleural de grande abondance.

**Chronologie:** infection au COVID-19 ayant entrainé une augmentation de la lymphocytose et du syndrome tumoral fait d’adénopathies et d’une splénomégalie supérieure à 10 cm ayant entrainé une première indication de traitement.

**Résultats cliniques:** l'examen pleuropulmonaire a retrouvé un syndrome d'épanchement pleural prédominant sur l'hémithorax gauche. Une ponction pleurale évacuatrice a été réalisée ramenant un liquide exsudatif séro-hématique lymphocytaire.

**Figure 1:**
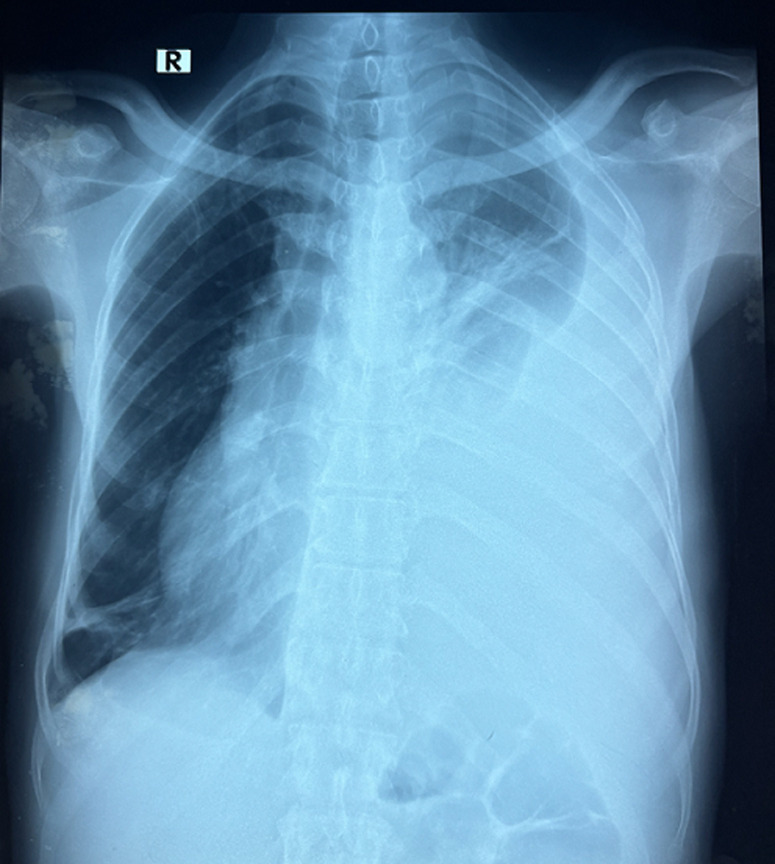
radiographie thoracique de face: pleurésie gauche de grande abondance révélant une tuberculose pleurale (étude de cas)

**Démarche diagnostique:** la radiographie thoracique de face a confirmé la présence d'une pleurésie gauche de grande abondance (Figure 1). L'interrogatoire a permis de retrouver une notion de contage tuberculeux dans la famille, sa sœur étant positive à la tuberculose. La recherche du bacille de Koch (BK) dans les crachats est revenue négative à l'examen direct de même que la culture. Le patient a ensuite bénéficié d'une biopsie pleurale qui a retrouvé des lésions granulomateuses avec nécrose caséeuse à l'examen anatomopathologique. La recherche par GeneXpert réalisée sur cette biopsie a pu confirmer la présence du complexe *Mycobacterium tuberculosis* de résistance indéterminée à la rifampicine.

**Intervention thérapeutique:** un drainage de l’épanchement pleural a permis d’obtenir une nette amélioration de la dyspnée. Un traitement antibacillaire a été initié sur une durée de 6 mois avec un traitement d'attaque de deux mois associant les quatre antibacillaires rifampicine (R), isoniazide (H), pyrazinamide (P), ethambutol (E), suivi de 4 mois sous RH.

**Figure 2:**
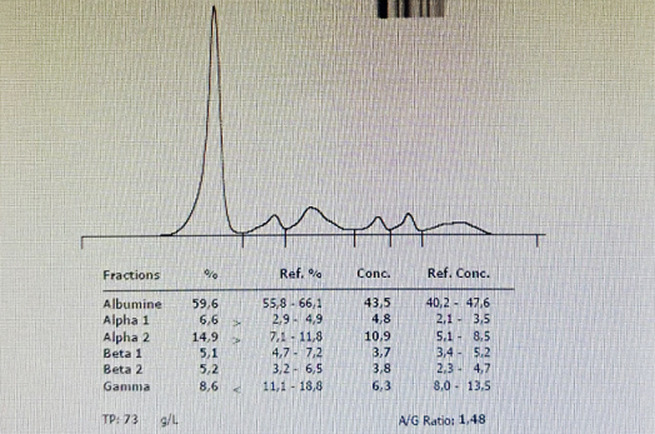
tracé d'électrophorèse des protéines sériques du patient montrant un syndrome inflammatoire associé à une diminution des gammaglobulines

**Suivi et résultats:** l’hyperlymphocytose a motivé l’hospitalisation du patient au service d’hématologie clinique. Le bilan biologique réalisé a montré une bicytopénie: Anémie à 8g/dl normochrome macrocytaire arégénérative ainsi qu'une thrombopénie à 97 000/mm3 avec une hyperleucocytose à 20 000/mm^3^ à prédominance lymphocytaire. Le reste du bilan a montré une élévation de la CRP à 48 mg/L. Un bilan hémolytique a retrouvé une élévation isolée des LDH à 580 mmol/l, sans élévation de la bilirubine. La ferritine a atteint 356 ng/mL avec une élévation modérée des transaminases à 2 fois la normale prédominant sur les ALAT. Une récente électrophorèse des protéines sériques a montré un syndrome inflammatoire modéré avec une hypogammaglobulinémie (Figure 2).

## Discussion

La récurrence des infections, comme illustré dans ce cas, est en faveur d'un dérèglement immunitaire qu'il convient d'explorer. La baisse des gammaglobulines observée en cas de LLC témoigne d'un affaiblissement des défenses immunitaires, ce qui rend le patient vulnérable à la survenue d'infections récurrentes, qu'elles soient virales ou bactériennes, notamment la tuberculose. Devant la multiplication des infections et les perturbations cliniques et biologiques, le diagnostic du SAM déclenché par le BK ne peut pas être exclu. Un myélogramme bien que non réalisé aurait été souhaitable pour appuyer cette hypothèse. L'agent causal le plus souvent retrouvé est d'origine virale, notamment l'EBV, bien que d'autres infections d'origine parasitaire ou bactérienne aient déjà été décrites, notamment l'association du SAM à la tuberculose dans sa localisation extrapulmonaire. Les bases physiopathologiques du SAM sont encore méconnues. L’activation des macrophages s’expliquerait par une production exagérée de cytokines pro-inflammatoires par les LT CD8 et les cellules NK d’où l’expression d’«orage cytokinique» [1].

**Figure 3:**
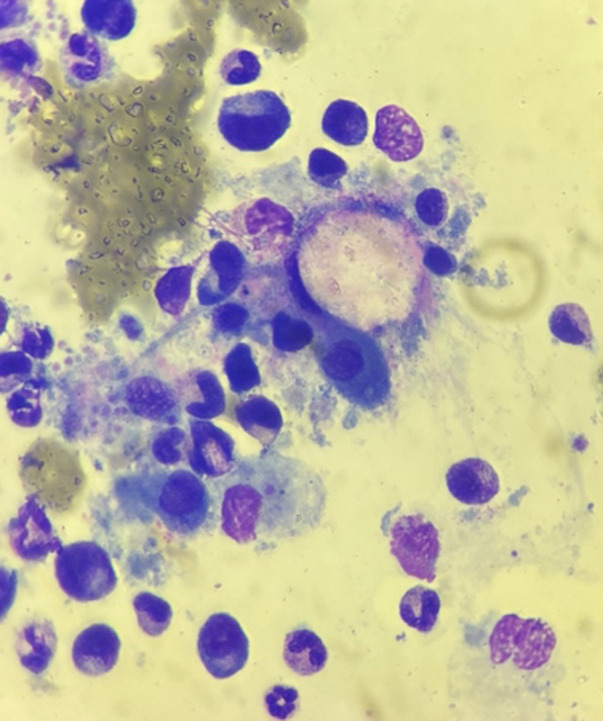
aspect cytologique d'hémophagocytose médullaire avec plusieurs macrophages activés chez un patient atteint de syndrome d’activation macrophagique (*1000)

**Figure 4:**
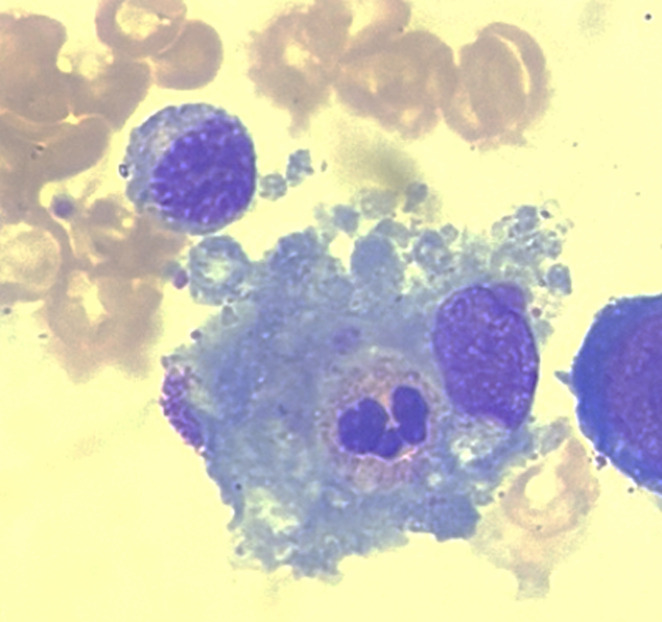
image cytologique d'un macrophage phagocytant un polynucléaire neutrophile et des plaquettes sur le myélogramme d'un patient atteint de SAM secondaire à une infection par l'EBV

**Figure 5:**
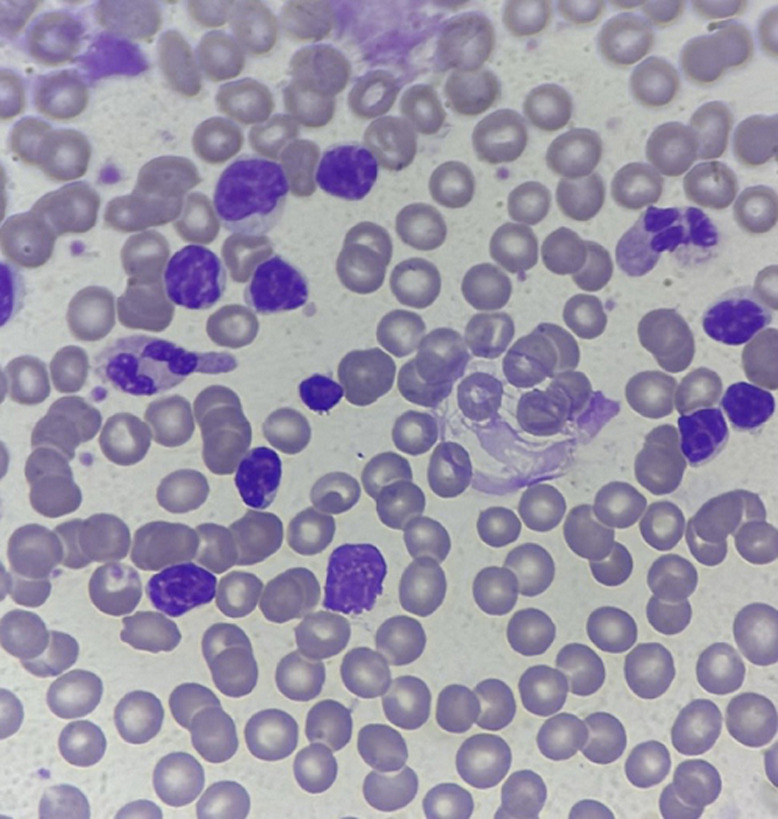
frottis sanguin typique de LLC avec une ombre de Gumprecht

Le diagnostic repose sur les critères d’hémophagocytose lympho-histiocytaire de 2004 (HLH-2004), incluant fièvre, cytopénies, hyperferritinémie, hypertriglycéridémie et hémophagocytose médullaire [5] résumés dans le tableau (Tableau 1) ci-dessous. La présence d'au moins 5 critères suffit pour affirmer le diagnostic de SAM. Le myélogramme est utile au diagnostic quand il retrouve de nombreux macrophages activés avec des images d´hémophagocytose (Figure 3,Figure 4). Dans le cas de notre patient, aucun myélogramme n'a été réalisé, ce qui complique le diagnostic de SAM. Contrairement à d'autres hémopathies, le diagnostic cytologique de LLC (Figure 5) sur sang périphérique suffit, ce qui rend le diagnostic médullaire inutile dans la majorité des cas. Sur le plan épidémiologique, la tuberculose pleurale constitue une forme extrapulmonaire fréquente dans les pays à endémie élevée, comme le Maroc. Une étude rétrospective conduite à Tétouan menée sur sept ans a retrouvé un taux d'incidence annuel de 127 cas pour 100 000 habitants, confirmant l'importance de la charge épidémiologique dans le nord du pays [6]. Enfin, une étude sur la tuberculose pulmonaire à Kénitra a mis en évidence une prédominance des formes pulmonaires, mais avec une proportion non négligeable d'atteintes extrapulmonaires, notamment pleurales [7].

**Tableau 1:** les critères de diagnostic du syndrome d'activation macrophagique

Fièvre	
	Splénomégalie
	Cytopénies affectant au moins deux lignées
**Hémoglobine < 9 g/dL**	
	Plaquettes < 100 000/mm^3^
	Polynucléaires neutrophiles<1000/mm^3^
**Hypertriglycéridémie et/ou hypofibrinogénémie**	
	Triglycérides > 3 mmol/L
	Fibrinogène < 1,5 g/L
**Hémophagocytose dans la moelle osseuse, rate ou ganglions lymphatiques**	
	Pas de néoplasie
	Activité des cellules natural Killer (NK) basse ou nulle
	Ferritinèmie> 500mg/L
	Récepteur soluble à l’IL-2 > 2400 UI/mL

SAM: syndrome d’activation macrophagique; NK: natural killer

Concernant la LLC, les données épidémiologiques marocaines restent limitées. Cependant, les hémopathies lymphoïdes représentent une part croissante des affections hématologiques diagnostiquées grâce aux progrès en immunophénotypage [8]. Quant au SAM, il reste rarement rapporté dans les registres nationaux, souvent décrit sous forme de cas isolés. La littérature rapporte très peu de cas associant SAM, LLC et tuberculose pleurale, ce qui confère à notre observation une valeur épidémiologique et scientifique notable. En ce qui concerne la littérature, l'association SAM-LLC est rarement rapportée. Une étude multicentrique réalisée sur 162 cas de SAM chez l'adulte [9] a montré que 56% des SAM étaient secondaires à des hémopathies malignes avec une prédominance des lymphomes T/NK et des lymphomes B pour plus de la moitié des cas. Les SAM secondaires à des leucémies aiguës ou chroniques étaient minoritaires. Nous n'avons retrouvé qu'une seule étude de cas réalisée sur une patiente âgée de 76 ans, suivie pour LLC en abstention thérapeutique, chez qui un SAM a été diagnostiqué et qui a révélé un lymphome hépatosplénique. Par ailleurs, l'association SAM-tuberculose est peu décrite. La dernière étude en date remonte à 6 ans, à propos de deux cas hospitalisés au service de pneumo-phtisiologie de l’Hôpital Moulay Youssef ayant développé un SAM sur une tuberculose multifocale pulmonaire avec atteinte médullaire [10].

Une étude rétrospective [3] réalisée en ce sens n´a permis de retrouver que 37 patients présentant cette association. En les classant en fonction de leurs comorbidités, sur les 37 patients associant le SAM à la tuberculose, seulement 3 étaient atteints d'hémopathie maligne dont deux de lymphomes. Un troisième cas de SAM a été rapporté chez une patiente de 56 ans en insuffisance rénale terminale compliquée d'un syndrome myélodysplasique. En cas de SAM associé, l'atteinte tuberculeuse la plus souvent rencontrée est extrapulmonaire comme retrouvée chez ce patient qui a contracté une tuberculose pleurale. Toute la difficulté du diagnostic de SAM associé à une LLC réside dans la similitude du tableau clinique où l’organomégalie est présente dans les deux cas. Les symptômes classiquement retrouvés dans un SAM étant semblables à ceux d’une LLC en progression, le diagnostic de SAM passe souvent inaperçu, d’autant plus lors d’infections associées. En effet, la LLC prédispose aux infections opportunistes, en particulier la tuberculose, et peut masquer ou mimer les symptômes du SAM. La triade observée dans ce cas illustre la complexité diagnostique : fièvre, cytopénies et inflammation peuvent être attribuées à l’une ou l’autre des pathologies. Une corrélation clinico-biologique étroite est donc indispensable pour distinguer le SAM d’une progression leucémique ou d’une infection isolée.

## Conclusion

Le syndrome d'activation macrophagique est une entité rare, encore mal documentée, potentiellement mortelle. Elle peut compliquer diverses pathologies, qu'elles soient néoplasiques, infectieuses ou auto-immunes. Cette observation illustre une triade diagnostique rare et complexe, associant leucémie lymphoïde chronique, tuberculose pleurale et syndrome d'activation macrophagique. Elle met en évidence la difficulté d'établir un diagnostic différentiel dans les pays à forte endémie tuberculeuse comme le Maroc, et souligne la nécessité d'une collaboration multidisciplinaire entre cliniciens, biologistes et anatomopathologistes afin d'optimiser la prise en charge et le pronostic. La documentation et la diffusion de tels cas demeurent essentielles pour améliorer la reconnaissance de ce syndrome afin d’affiner sa compréhension épidémiologique à l’échelle nationale. La recherche d'infections associées, notamment la tuberculose dans notre contexte, n'est pas à écarter malgré les similitudes cliniques avec la LLC. Comme en témoigne cette étude de cas, les sujets atteints de LLC sont particulièrement vulnérables aux infections. Compte tenu de notre contexte d'endémie, nous insistons sur l'intérêt du dépistage précoce de la tuberculose en commençant par la recherche et le traitement des cas contagieux dans l'entourage afin d'améliorer le pronostic vital des patients immunodéprimés. Le partage et la documentation des cas diagnostiqués à l’échelle nationale demeurent essentiels afin d’améliorer la reconnaissance de ce syndrome et affiner sa compréhension épidémiologique locale.
